# Embolic retinal and choroidal vascular occlusion after peribulbar triamcinolone injection: A case report

**DOI:** 10.1097/MD.0000000000010467

**Published:** 2018-04-27

**Authors:** Gang Li, Dongdong Xu, Zhirou Hu, Hui Li

**Affiliations:** a Department of Ophthalmology, Peking Union Medical College Hospital, Chinese Academy of Medical Sciences, Beijing; b Department of Ophthalmology, The First Affiliated Hospital of Zhengzhou University, Zhengzhou University, Zhengzhou, China.

**Keywords:** central retinal artery occlusion, peribulbar injection, thyroid-associated ophthalmopathy, triamcinolone acetonide

## Abstract

**Rationale:**

Retinal and choroidal vascular occlusion is a vision-threatening complication of therapeutic injections in the facial region. The early identification and early treatment are necessary to reduce the risk of harm to the patient.

**Patient concerns:**

We report an extremely rare case of embolic retinal and choroidal vascular occlusion after peribulbar triamcinolone injection in a patient with thyroid-associated ophthalmopathy.

**Diagnoses:**

Central retinal artery occlusion.

**Interventions:**

First, we performed a fundus examination in the patient. Triamcinolone embolus was observed in both retinal and choroidal vessels. Anterior chamber paracentesis and ocular massage combined with venous injections of alprostadil and Ginaton as well as an acupoint injection of compound anisodine were performed immediately. Sublingual glyceryl trinitrate and intraocular pressure-lowering drugs were also administered. Fundus autofluorescence, optical coherence tomography-angiography, fundus fluorescein angiography (FFA), and indocyanine green angiography (ICGA) were also conducted to evaluate the patient's condition.

**Outcomes:**

One month after the onset of the situation, the triamcinolone embolus had disappeared. The retinal edema and retinal blood perfusion were also improved. The patient's visual acuity had recovered from inexact light perception to 0.02.

**Lessons:**

Embolic retinal and choroidal vascular occlusion is vision-threatening disease. Measures such as careful aspiration before injecting in the facial region must be taken to avoid such complications.

## 1 Introduction

Thyroid-associated ophthalmopathy (TAO), which is the most common extrathyroidal manifestation of Graves’ disease, is an orbital disease that usually manifests in thyroid dysfunction. Repeated peribulbar injections of triamcinolone acetonide are an effective treatment for moderate-to-severe TAO, of which the most frequent side-effect is increased intraocular pressure.^[1]^ Vision-threatening complications such as embolization of the retinal and choroidal circulation may follow therapeutic injections around the eyes, nose, and ears.^[2–4]^ This article reports a rare case of acute vision loss with combined retinal arteriolar occlusion and choriocapillaris following a periocular injection of triamcinolone acetonide.

## 2 Case presentation

**Figure 1:**
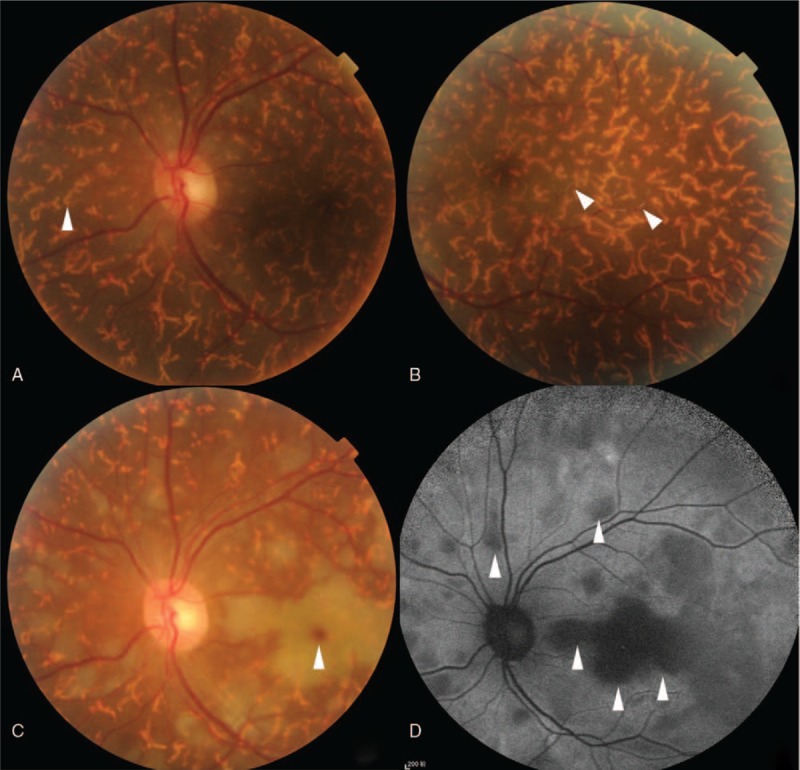
Fundus photograph of the left eye showing the deposition of triamcinolone particles in the retinal terminal arteries (white arrow in [B]) and choriocapillaris (white arrow in [A]) when the patient suffered vision loss. The fundus photograph also shows ischemic retinal whitening with a cherry red spot (white arrow in [C]). Fundus autofluorescence shows hypoautofluorescence of the nonperfused retina (white arrow in [D]).

**Figure 2:**
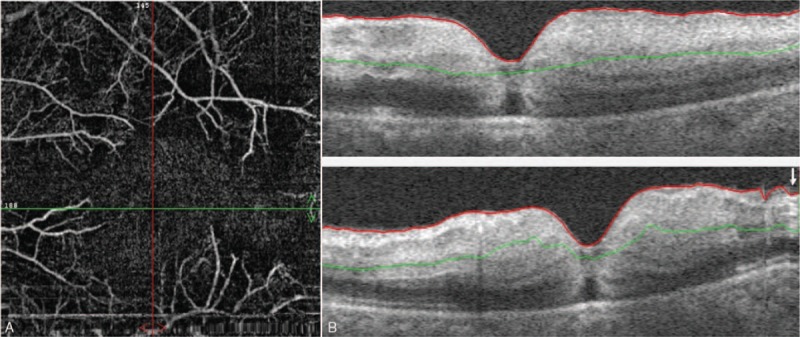
Vascular density in the left eye is significantly decreased compared with the right eye. The retinal capillary network is discontinuous, and a capillary drop out is noticeable (A). The thickness of the retinal neurosensory layer is increased (B).

**Figure 3:**
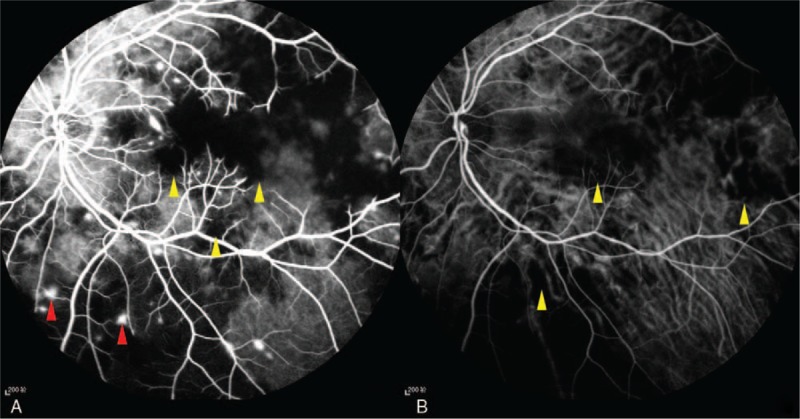
Fundus fluorescein angiography (FFA) showing retinal arteriolar filling defects in the paramacular area (yellow arrow in [A]). The obvious fluorescein leakage is caused by ischemia of the retina (red arrow in [A]). Indocyanine green angiography (ICGA) shows hypofluoresence area in the choroid, suggesting areas of choroidal hypoperfusion due to triamcinolone emboli blocking the choroidal capillaries (yellow arrow in [B]). FFA = fundus fluorescein angiography, ICGA = indocyanine green angiography.

A 48-year-old female patient with moderate-to-severe TAO was given the fourth superior-nasal periocular injections of 20 mg triamcinolone acetonide around her eyes. Approximately 20 minutes after the injection, the patient described acute loss of vision and feelings of swelling and pain in her left eye. Ocular examinations showed inexact perception of light in the left eye with obvious subconjunctival hemorrhage and chemosis. The left pupil was dilated, and both direct and consensual pupillary light reflexes were insensitive. A fundus examination was conducted immediately, which showed deposits of whitish material in the temporal retinal vessels and subretinal yellowish white strips in the left eye (Fig. 1A and B). A day later, fundus photography revealed ischemic retinal whitening with a cherry red spot (Fig. 1C). Fundus autofluorescence showed hypoautofluorescence of the nonperfused retina (Fig. 1D). Optical coherence tomography-angiography (OCT-A) was performed at the area of the posterior pole. The OCT-A scan showed that the vascular density in the left eye was significantly decreased compared with the right eye. The retinal capillary network was discontinuous, and capillary dropout was noticeable. The thickness of the retinal neurosensory layer was increased (Fig. 2A and B). Fundus fluorescein angiography (FFA) and indocyanine green angiography (ICGA) were also performed. Areas of hypofluoresence in the choroid were suggestive of choroidal nonperfusion areas. Retinal arteriolar filling defects were present in the paramacular area, and fluorescein leakage was obvious (Fig. 3A and B). All observations were thought to be associated with the blockage of multiple choroidal vessels and the retinal arteries by triamcinolone acetonide particles.

Anterior chamber paracentesis and ocular massage were performed in the patient immediately in addition to venous injections of alprostadil and Ginaton and acupoint injection of compound anisodine. Antiglaucoma drugs such as carteolol, brinzolamide, and Alphagan eye drops were administered to the patient's left eye. Sublingual glyceryl trinitrate was administered immediately after the diagnosis.

**Figure 4:**
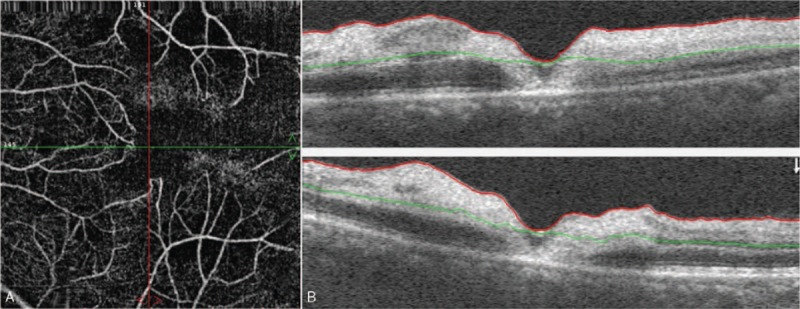
OCT-A showing that vascular density (A) in the left eye is increased and the retinal neurosensory layer edema is improved (B).

**Figure 5:**
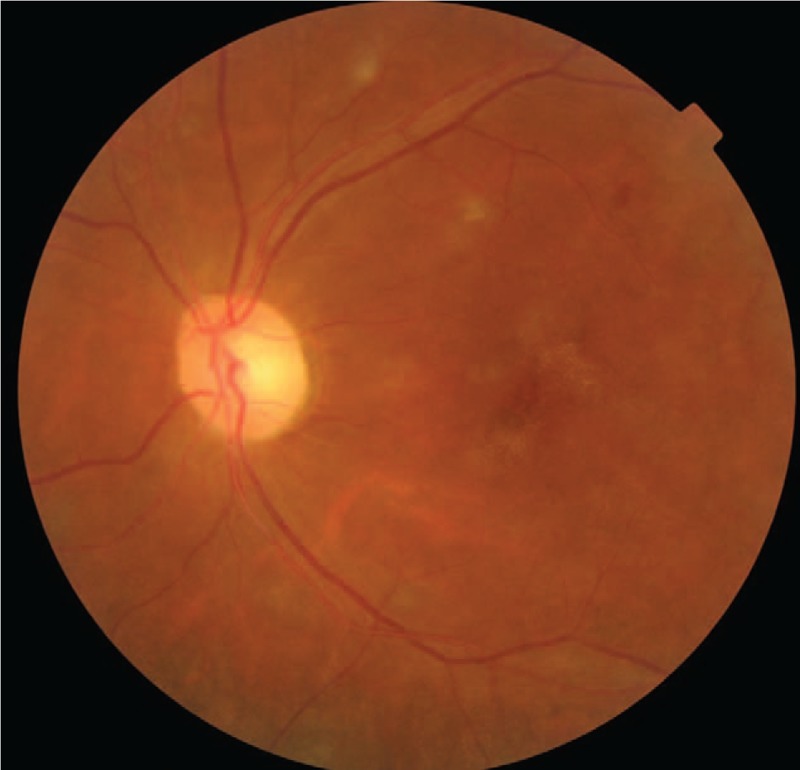
Follow-up showing that the crystals of triamcinolone acetonide in the retinal vessels and choroidal capillaries had disappeared.

The patient was followed-up for more than 1 month. OCT-A was repeated 12 days after the first visit. The vascular density in the left eye was increased, and the retinal neurosensory layer edema was improved (Fig. 4). At 40 days, fundus photography showed the absorption of triamcinolone particles (Fig. 5) and her visual acuity improved from inexact light perception to 0.02. At the last visit 26 months later, the patient‘s visual acuity recovered to 0.05.

## 3 Discussion

Central retinal artery occlusion (CRAO)is an ophthalmic emergency because it is a severe threat to vision. The incidence of CRAO is approximately 1 in 100,000 people, which accounts for 1 in 10,000 ophthalmological outpatient visits. Most cases of RAO were reported to be caused by thromboembolic disease due to atherosclerosis.^[5]^ In 1978, Ellis^[6]^ reported a case of CRAO after retrobulbar corticosteroid injection. After intralesional steroid injection in the craniofacial region RAO was also reported in recent years.^[3,7–10]^ The mechanisms were explained as follows. First, because there are diffuse anastomoses in the facial arterial system, facial injections might cause retrograde embolization of the ophthalmic or central retinal arteries.^[3,8]^ Second, the inattentive intra-arterial injection of the glucocorticoids and the consequent retrograde flow of glucocorticoid suspension could cause vascular occlusion.^[10]^ Third, the particle size in glucocorticoid suspension ranges from 1 to 1000 μm.^[11]^ The mean caliber of the retinal arteriole is 144.1 ± 14.4 μm (SD), and the mean caliber of the venular is 214.0 ± 22.2 μm.^[12]^ The reason for the embolism might be that the diameter of some particles is larger than the caliber of the blood vessels in the retina. Fourth, the forceful injection might have promoted the embolism in the ophthalmic artery.^[9]^

In this study, the patient was a 48-year-old female who developed an acute onset of CRAO after a supposed peribulbar triamcinolone injection for TAO. To the best of our knowledge, this is the first documented case of CRAO after a peribulbar triamcinolone injection for TAO. The inattentive intra-arterial injection of triamcinolone could have caused CRAO in this patient. A fundus examination was performed as soon as the patient presented with severe vision loss. Yellowish white embolic material was observed to be deposited in both small retinal vessels and choridal vessels. Glucocorticoid embolus in retinal vessels was reported previously, but not in choridal vessels.^[3,6]^ Thus, the present case might be the first reported in which triamcinolone embolus was seen in choridal vessels. Retinal edema and cherry red spot in macula appeared 24 hours after the triamcinolone injection. It reminds us that fundus examination should be performed if a patient suffer from acute vision loss after triamcinolone injection in facial region and central retinal artery occlusion should be seriously considered if embolic material in retinal and choroidal vessel were observed, because typical cherry red spot appeared 24 hours after CRAO onsets in our case. In the patient, fundus autofluorescence showed hypoautofluorescence in the nonperfused region in the retina. The etiology of the hypoautofluorescence might be that the inner retinal swelling blocked the retinal pigment epithelium autofluorescence^[13]^ or the oxygenated and deoxygenated nicotinamide adenine dinucleotide (NADH), and the flavin adenine dinucleotide (FAD) emission spectra produced the phenomenon of low fluorescence.^[14]^ OCT and OCT-A are noninvasive examinations that are conducted to evaluate retina and retinal vessel density. In this patient, OCT and OCT-A showed retinal neurosensory layer edema and significantly decreased vascular density. Discontinuous retinal capillary network and capillary dropout were also observed in the OCT-A, which were caused by retina ischemia. FFA and ICGA were also performed in this patient. Areas of hypofluoresence were detected in the choroid, indicating nonperfusion areas. Retinal arteriolar filling defects in the paramacular area and obvious fluorescein leakage were observed. Anterior chamber paracentesis, ocular massage, venous injections of alprostadil, and Ginaton as well as acupoint injections of compound anisodine, sublingual glyceryl trinitrate, and antiglaucoma drugs were administered as soon as the diagnosis was made. During the follow-up, the OCT and OCT-A scan showed that retinal edema and retinal vessel density were improved at one month in, which indicated that these examinations were useful in patients with CRAO. The patient's visual acuity improved from the inexact perception of light to 0.02 after treatment was administered without delay.

To the best of our knowledge, this study is the first to describe the medical record of a patient with CRAO caused by after retrobulbar corticosteroid injection. The images of fundus photo, FFA, ICGA, OCT, and OCT-A were well recorded, and the treatment and the prognosis were well documented. These findings could provide significant information for ophthalmologists in evaluating similar patients.

## 4 Conclusion

Embolic retinal and choroidal vascular occlusion is a vision-threatening complication of therapeutic injections in the facial region. In patients with significant visual loss after facial triamcinolone injection, CRAO is strongly indicated if embolic material in the retinal and choridal vessels is observed. If treatment is delayed, typical manifestations of CRAO, such as cherry red spots, might occur, and the optimal intervention window might be missed. To avoid this complication, utmost care should be taken and careful aspiration must be performed before injecting a steroid suspension in the facial region.

## Acknowledgment

The authors thank the patient who participated in the present study for providing written permission to publish this case report.

## Author contributions

**Conceptualization:** Gang Li, Dongdong Xu, Hui Li.

**Data curation:** Gang Li, Dongdong Xu, Zhirou Hu, Hui Li.

**Formal analysis:** Hui Li.

**Funding acquisition:** Hui Li.

**Gang Li & Dongdong Xu** carried out the entire procedure including performed examination, literature search, drafted the manuscript, revised submitted the manuscript, these two authors contributed equally to this article. **Hui Li** conceived of the diagnosis and treatment of this patient, coordinated and participated in the entire process of drafting and revised the manuscript. **Zhirou Hu** contributed to data collection and revision the manuscript. All authors have contributed significantly. All authors read and approved the final manuscript

**Investigation:** Gang Li, Dongdong Xu, Zhirou Hu, Hui Li.

**Methodology:** Gang Li, Dongdong Xu, Zhirou Hu, Hui Li.

**Project administration:** Gang Li, Dongdong Xu, Hui Li.

**Resources:** Gang Li, Dongdong Xu, Hui Li.

**Software:** Gang Li, Dongdong Xu, Zhirou Hu, Hui Li.

**Supervision:** Gang Li, Dongdong Xu, Hui Li.

**Validation:** Gang Li, Dongdong Xu, Hui Li.

**Visualization:** Gang Li, Dongdong Xu, Zhirou Hu, Hui Li.

**Writing – original draft:** Gang Li, Dongdong Xu, Hui Li.

**Writing – review & editing:** Gang Li, Dongdong Xu, Hui Li.
